# Immune-mediated entities of (primary) focal segmental glomerulosclerosis

**DOI:** 10.1007/s00441-021-03454-3

**Published:** 2021-04-27

**Authors:** Fabian Braun, Inka Homeyer, Nada Alachkar, Tobias B. Huber

**Affiliations:** 1 III. Department of Medicine, University Medical Center Hamburg-Eppendorf, Martinistraße 52 , 20246 Hamburg, Germany; 2 Division of Nephrology, Department of Medicine, The Johns Hopkins University School of Medicine, Baltimore, MD USA

**Keywords:** Primary focal segmental glomerulosclerosis, Podocyte, Immune cell, Immune epithelial interaction, Soluble factor

## Abstract

Focal segmental glomerulosclerosis (FSGS) represents a glomerular scar formation downstream of various different mechanisms leading to podocytopathy and podocyte loss. Recently, significant advances were made in understanding genetic factors, podocyte intrinsic mechanisms, and adaptive mechanisms causing FSGS. However, while most cases of nephrotic FSGS are being treated with immunosuppressants, the underlying immune dysregulation, involved immune cells, and soluble factors are only incompletely understood. Thus, we here summarize the current knowledge of proposed immune effector cells, secreted soluble factors, and podocyte response in immune-mediated (primary) FSGS.

## Introduction

Focal segmental glomerulosclerosis (FSGS) is one of the leading glomerular causes of end-stage kidney disease (ESKD). The FSGS definition still mostly relies on the characteristics of the sclerotic lesion, which is the consequence of multiple different underlying etiologies and podocytopathies. To better reflect the pathophysiology, Kopp et al. recently proposed six entities of podocyte injury: association with low nephron number or increased body mass, genetic causes, infectious agents, pregnancy-related VEGF inhibition, drugs, as well as immunological and soluble factors (Kopp et al. 2020). In the following, we will use the term primary FSGS for the presumably (soluble factor) immune-mediated form of FSGS.

The main hypothesis—one or multiple circulating plasma factors leading to podocyte damage in primary FSGS—is supported by several observations. Approximately 40–50% of primary FSGS patients undergoing kidney transplantation develop a recurrence of FSGS in the allograft (Hickson et al. 2009; Maas et al. 2013a). Onset of recurrence of FSGS can occur within hours after transplantation (Francis et al. 2016). Further support to this was given by a case report, depicting the resolution of FSGS recurrence in a kidney allograft explanted from a patient with primary FSGS and subsequent implantation into a patient suffering from diabetic nephropathy (Gallon et al. 2012) and the transient development of proteinuria in a newborn child born from a mother with primary FSGS (Kemper et al. 2001). Additionally, the observation that plasma exchange and immunoadsorption reduce proteinuria in many cases of primary or recurrent FSGS, possibly due to removal of the pathological factor(s) and thus decrease of FSGS activity, strengthens the potential contribution of plasma intrinsic factors to the development of FSGS (Zimmerman 1985; Dantal et al. 1994).

The causative permeability factor(s) of primary FSGS as well as its/their origin, however, remain elusive. Several findings point to a key contributory role of the immune system. Among these indications are the resolution of idiopathic nephrotic syndrome upon measles infection, the successful treatment with steroids, and other immune-modulating targets as well as increased levels of specific cytokines in FSGS patients’ plasma and urine (Shalhoub 1974; Suranyi et al. 1993).

This review aims to give a concise overview of the current knowledge of the potential effector cells and released factors of the immune system involved and the epithelial reaction to these, delineating primary and recurrent FSGS as a disease based on immune-epithelial interactions.

## Potential effector cells in primary FSGS

Despite the compelling clinical evidence that the immune system plays a decisive role in the development of primary FSGS, the precise characterization of the immune phenotype in primary FSGS is largely unknown. Likewise, it remains unclear, whether this role is exerted through direct cellular interaction between immune and epithelial cells or through secreted factors over greater distances. This is in part due to a lack of clinical stratification and, for pediatric cases of nephrotic syndrome, missing histological stratification as kidney biopsies are obtained in a minimal portion of nephrotic children. The current knowledge, therefore, mostly stems from preclinical models or heterogenous patient cohorts with limited potential to deduce conserved pathological mechanisms.

## T cells

Initially, the circulating permeability factor was suggested to be released by T cells (Shalhoub 1974) based on clinical observations like the remission of nephrotic syndrome upon measle infection, immunosuppressive therapy, e.g., steroids or cyclophosphamide, and the lack of evidence for a humoral antibody response (Shalhoub 1974). Furthermore, diagnosis of hematological neoplasia in some cases coincided with nephrotic syndrome. Interestingly, treatment of these conditions also resolved proteinuria, suggesting a relationship between dysregulated lymphocytes and the development of nephrotic syndrome (Shalhoub 1974).

Investigation of contributing cells to primary FSGS pathogenesis in animal models also implied participation of T cells (Berre et al. 2005). After kidney transplantation, Buffalo/Mna rats develop a spontaneous recurrence of glomerulopathy, mimicking recurrent pFSGS. Vice versa, transplantation of a kidney from a Buffalo/Mna rat into wild-type rats ameliorates the pathological conditions (Berre et al. 2002). Analysis of the immune processes in early stages of the spontaneous glomerulopathy revealed macrophages and T cells infiltrating into the kidney. Despite a minor T cell infiltrate, an increase in Cβ TCR transcription products was observed. Bulk RNA analysis of kidney samples to assess the T cell infiltrate revealed a cytokine transcript expression profile prompting to an involvement of Th2 cells, while a downregulation of Th1 cell cytokines was detected (Berre et al. 2005). This shift towards a Th2 phenotype was also observed in children with nephrotic syndrome (Kemper et al. 2005).

Besides Th2 cells, regulatory T cells (T_reg_ cells) were suggested to play a role in controlling the pathogenesis of FSGS. This assumption was supported by subsequent in vivo studies with investigating the antiproteinuric effect of LF15-0195 treatment, a 15-deoxyspergualin (DSG) derivate (Berre et al. 2009). DSG is an immunosuppressive drug, which acts by inhibition of T and B lymphocyte differentiation, as well as blockade of macrophage function and activation (Berre et al. 2009). LF15-0195 treatment in Buffalo/Mna rats resulted in a decrease of proteinuria to normal levels, as well as an amelioration of histological lesions. FACS analysis of blood and spleen cells revealed no change in the frequency of T cells, B cells, NK cells, or monocytes, but an increased frequency of CD4+ CD25+ T cells in treated proteinuric animals (Berre et al. 2009). A total of 65.4% of CD4+ CD25+ T cells in the spleen and 82.3% in the peripheral blood were FoxP3 positive, a master regulator of T_reg_ cells (Steinmetz et al. 2011). Cell transfer studies showed that these LF15-0195-stimulated CD4+ CD25+ T cells were able to induce remission in recipient proteinuric rats suggesting that T_reg_ cells might play a role in the pathogenesis of FSGS.

Investigating renal biopsies from 38 pediatric patients, 15 classified as FSGS, and 23 as minimal change glomerulopathy (MCG), Benz et al. detected a higher number of interstitial CD3+ T cells and macrophages in patients with FSGS (Benz et al. 2010). The number of FoxP3+ T cells was significantly lower in FSGS, MCG, and steroid-dependent patients compared with controls and located exclusively to the tubulointerstitium but not the glomeruli (Benz et al. 2010).

FACS analysis of peripheral blood mononuclear cells (PBMC) from primary nephrotic syndrome (PNS) patients revealed decreased levels of T_reg_ cells and increased levels of Th17 cells (Shao et al. 2009). Also, the frequency of mRNA transcripts of Th17 cell-related factors, such as IL-17, IL-23P19, and RORc, was increased in PBMC from patients with PNS. IL-17 expression in correlating kidney biopsies revealed the highest expression in FSGS cases exhibiting tubular-interstitial injury. Positive immunostaining for IL-17 was detected in the glomerular and tubular compartment (Shao et al. 2009). These results were correlated to higher frequencies of circulating Th17 cells and mRNA levels of Th17 cell-associated factors in children with PNS (Wang et al. 2013). Evaluation of kidney biopsies showed higher renal expression rates of IL-17 and IL-1β in FSGS samples. Furthermore, a positive correlation between IL-17, IL-1β, and IL-16 with glomerulosclerosis was observed (Zhai et al. 2020).

Taken together, these studies suggest a role of T cell subsets as mediators of primary FSGS, while the precise mechanisms and specific subgroups remain unknown.

## Macrophages

Intracapillary accumulation of foam cells often precedes the characteristic FSGS lesions, especially in the cellular variant (D’Agati et al. 2004). Macrophages accumulate in glomerular endocapillary sites, where uptake of low-density lipoproteins leads to differentiation into foam cells.

The frequency of monocyte-macrophage lineage cells and the expression of macrophage-associated factors were found to be higher in the kidney infiltrate of Buffalo/Mna rats (Berre et al. 2005). Macrophage-associated cytokine transcripts, including TNFα, IL-12, IL-6, and IL-1, were highly expressed in proteinuric rats. Concordantly with an increase of the monocyte/macrophage cell population, significantly elevated levels of TNFα mRNA transcripts preceded the onset of proteinuria and histological lesions by several weeks. Corresponding to these animal studies, significantly higher numbers of interstitial CD68+ macrophages were detected in kidney samples from pediatric FSGS patients (Benz et al. 2010) while glomerular macrophage cell count only showed a tendency towards higher frequency in FSGS biopsies (Benz et al. 2010).

While Le Berre et al. suggest a contribution of macrophages in the very early stages or even the initiation of FSGS development, further investigations proposed a role of macrophages in FSGS progression after occurrence of podocyte injury (Berre et al. 2005; Hara et al. 2015). Investigating the underlying mechanisms of glomerular foam cell formation in FSGS, using NEP25 (podocyte-selective injury), LDLR^−/−^ (hypercholesterolemia) and NEP25/LDLR^−/−^ mice, neither hypercholesterolemia nor podocyte injury alone was sufficient to induce macrophage/foam cell infiltration to glomeruli. Occurrence of glomerular lipid deposits and glomerular CD68+ macrophages and foam cells were highest in adriamycin-treated LDLR^−/−^ mice with high-fat diet (ADR+ HFD/LDLR^−/−^) (Hara et al. 2015).

Subsequent studies with NEP25/LDLR^−/−^ mice indicated that podocyte-specific injury in context with hypercholesterolemia leads to glomerular lipid peroxidation and formation of lysophosphatidylcholine (LPC) 16:0 and 18:0, affecting the expression of chemokines and adhesion molecules essential for macrophage attraction and migration. These findings raised the hypothesis that podocyte injury in the presence of hypercholesterolemia leads to lipid deposition and peroxidation with formation of specific LPC. LPC may play a role in modulating glomerular resident cells, which eventually results in glomerular macrophage infiltration and foam cell formation (Hara et al. 2015).

Hence, the direct contribution of macrophages and monocytes to FSGS and the specific timepoint of action in promoting lesion formation are in need for further clarification.

## B cells

The hypothesis of B cell involvement in the pathogenesis of primary FSGS was proposed after successful treatment of FSGS patients with rituximab, a CD20 antibody, leading to B cell depletion (Nozu et al. 2005; Pescovitz et al. 2006). Further studies on the effect of rituximab in FSGS revealed an amelioration for a subset of FSGS patients (Araya and Dharnidharka 2011). While the effect of rituximab on B cells is well studied, broader effector mechanisms were suspected revealing a direct effect on podocytes by preventing the downregulation of sphingomyelin-phosphodiesterase-acid-like-3b (SPMDL-3b) and stabilizing the podocyte actin-cytoskeleton, potentially resulting in less podocyte effacement (Fornoni et al. 2011). These results questioned the contribution of B cells in the FSGS pathogenesis and put emphasis on an additional mode of action of rituximab besides B cell depletion.

Nevertheless, analysis of the immune infiltrate of kidney biopsy samples from children with idiopathic nephrotic syndrome revealed a significantly higher number of glomerular CD20 + B cells in FSGS patients (Benz et al. 2010). Beneficial effects of rituximab treatment on proteinuria, however, were also observed in the absence of interstitial or glomerular B cells, questioning a potential pathogenic role of B cells in FSGS (Benz et al. 2010). Still, the next-generation anti-CD20 antibody ofatumumab showed the potential of inducing proteinuria remission in a rituximab-resistant case, indicating the need for further investigations on the effect of anti-CD20 antibodies in nephrotic syndrome.

## Cells affected by FSGS

While the cells initiating the development of FSGS are not fully identified yet, renal resident cells and processes involved in the formation of glomerular lesions in FSGS are partially understood. Different developmental FSGS stages precede the full manifestation of glomerular sclerosis. Kriz et al. described a coherent sequence of events in detail (Kriz 2002). Following an initial insult podocyte loss leads to exposure of bare glomerular basement membrane (GBM). Remaining podocytes react to this by hypertrophy (Puelles et al. 2019). When this compensatory mechanism is overcome, denuded capillaries can get in contact with the parietal epithelial cells (PEC) lining Bowman’s capsule promoting adhesion of PEC to the glomerular tuft (Kriz 2002; Shankland 2006). Subsequently, larger parts of the glomerulus develop sclerosis in the end resulting in obliteration of the capillary network and loss of the nephron. This sequence characterizes the secondary processes in lesion formation in most cases of FSGS—one exception being FSGS of the collapsing type. However, the profiling of early events in primary FSGS, arguably the most suitable timepoint for therapeutic intervention, has been hampered by heterogenous patient cohorts and unspecific preclinical models. While it is commonly accepted that podocyte injury is the initial step of FSGS pathogenesis, the focus has recently broadened to the contribution of other residential kidney cells.

## Podocytes

Before severe podocyte injury and glomerulosclerosis develop, podocyte loss to a specific threshold is compensated by hypertrophy of the remaining glomerular epithelial cells. Our group, among others, has suggested podocyte hypertrophy mediated by the mTOR-signaling pathway to prevent PEC activation and glomerulosclerosis. This beneficial compensatory response can turn into a pathological process when extensive mTOR-dependent hypertrophy is not able to counterbalance the loss, leading to an exacerbation of it (Gödel et al. 2011; Fantus et al. 2016; Grahammer and Huber 2016; Zschiedrich et al. 2017; Puelles et al. 2019).

There is limited knowledge about the direct effects of immune factors on podocytes mostly stemming from in vitro experiments in different glomerular pathologies (Milas et al. 2019). Still, some knowledge can be gained to extrapolate a link between the immune system and podocytes in primary FSGS. Application of recombinant murine IL-17 on murine podocytes promoted podocyte apoptosis in a dose- and time-dependent manner and a reduction of podocalyxin expression (Wang et al. 2013) and aggravated podocyte damage in nephrotic children (Zhai et al. 2020). Podocytes in vitro express the IL2-R and its activation causes damage through increased autophagy (Stewart et al. 2020) and interleukin 4 overexpression in mice induces proteinuria and CKD (Kim et al. 2017). On the other hand, IL-9 found to exert protective effects in adriamycin-induced glomerulopathy (Lin et al. 2020; Xiong et al. 2020).

Matsusaka et al. generated transgenic NEP25 mice, expressing human CD25 (hCD25) selectively in podocytes, leading to irreversible podocyte injury upon injection of recombinant immunotoxin LMB2 (Matsusaka et al. 2005). Selective injury to podocytes promoted global or segmental glomerulosclerosis, affecting endothelial cells, parietal and tubular epithelial cells, as well as mesangial cells (Matsusaka et al. 2005). Other animal models which allow for selective podocyte damage, consecutively leading to development of proteinuria and eventually glomerulosclerosis, are, e.g., Thy 1.1 transgenic mice as well as mice and rats carrying the transgenic human diphtheria toxin receptor on podocytes (Assmann et al. 2002; Wharram et al. 2005). Wharram et al. showed that podocyte loss of at least 20% is required for the development of glomerulosclerosis, while podocyte depletion < 20% was unable to initiate glomerulosclerosis and could be restored (Wharram et al. 2005). Our group further specified this limit to be 24% in several mouse models for glomerular injury and human diseases (Puelles et al. 2019).

Once podocytes undergo injury independent of the etiology, conserved mechanisms have been recognized (Rinschen et al. 2018). The actin cytoskeleton, crucial for maintenance of the specific shape of podocytes, undergoes changes, resulting in morphological alternations, characteristically cell body attenuation, loss of the slit diaphragm, and foot process effacement (Kriz et al. 1998; Jefferson and Shankland 2014; Grahammer et al. 2016; Schell et al. 2017; Schell and Huber 2017). These changes in cell shape and adhesion precede podocyte detachment from the glomerular basement membrane leading eventually to a reduced podocyte number (Jefferson and Shankland 2014; Braun et al. 2016; Schell et al. 2018; Puelles et al. 2019). Besides the development of effacement as the earliest feature of FSGS and its recurrence post-transplant, there are other aspects emphasizing podocyte damage as the initial step in primary FSGS development (Chang et al. 2012; Alachkar et al. 2013).

While there are attempts to interfere in these later stages of podocyte loss in glomerular sclerosis (Schiffer et al. 2015), a better understanding of the early injury patterns and mechanisms most likely hold the greatest premise for therapeutic interventions. This is of particular importance since the potential of podocyte regeneration from progenitor cells seems to be limited at best (Poulsom et al. 2001; Appel et al. 2008; Ronconi et al. 2009; Meyer-Schwesinger et al. 2011; Grahammer et al. 2013; Pippin et al. 2013; Wanner et al. 2014).

## Parietal epithelial cells

While mature differentiated podocytes inhere little ability of proliferation and regeneration under physiological conditions (Wanner et al. 2014), mature parietal epithelial cells (PECs) are able to proliferate (Jefferson and Shankland 2014). Ambiguous opinions have been expressed on the role of PECs in FSGS pathogenesis. Despite the aforementioned assumption of PECs as potential progenitor cells for podocytes, PECs seem to promote lesions when activated, leading to progression of FSGS (Barisoni et al. 1999; Appel et al. 2008; Smeets et al. 2009, 2011; Fatima et al. 2012).

Using cell lineage tracing, it was reported that activated PECs are involved in the formation of sclerotic lesions (Smeets et al. 2009, 2011). Upon an insult like podocytopenia, PECs can get activated and form an adhesion between Bowman’s capsule and glomerular tuft (Smeets et al. 2011). Invasion of activated PECs to the glomerular tuft and subsequent matrix deposition of Bowman’s capsule occurs, leading to progression of sclerosis (Smeets et al. 2011).

PEC activation and glomerular tuft adhesion seem to occur at very early stages of FSGS development, representing a potential tool for diagnosis using CD44 expression as a PEC activation marker to differentiate FSGS and minimal change disease (Fatima et al. 2012; Smeets et al. 2014).

Beside the participation of classical flat PECs lining Bowman’s capsule, two other subpopulations of PECs were reported to participate in the development of FSGS, especially in glomerular tip lesions (Kuppe et al. 2019). Proximal tubular epithelial-like parietal epithelial cells (cuboidal PECs) reside at the most proximal part of the proximal tubule. Additionally, intermediate PECs were identified, lining the tubular orifice between cuboidal and flat PEC. Both PEC subgroups were found to participate in the formation of sclerotic lesions in mice and were activated more easily than flat PEC. Analysis of biopsies from recurrent FSGS patients with tip lesions revealed in 90% positivity for intermediate PEC markers, suggesting a contribution of this cell type in sclerotic lesion formation (Kuppe et al. 2019).

## Endothelial cells

While endothelial cell integrity remains intact in earlier stages of FSGS, consecutive progression of glomerulosclerosis leads to endothelial injury (Jefferson and Shankland 2014). The late impairment of endothelial cells suggests a secondary involvement in the pathogenesis of FSGS.

Eremina et al. suggested a reciprocal influence of vascular endothelial growth factor A (VEGF-A)-producing podocytes and endothelial cells as VEGF-A plays an important role in the maintenance of the fenestrated endothelium, which is impaired upon podocyte injury (Eremina et al. 2003, 2008).

The interaction of endothelin-1 (EDN1)/EDN1 receptor type 1 (EDNRA) could also be a decisive contributor in podocyte-endothelial cell crosstalk (Daehn et al. 2014). In adriamycin nephropathy, EDN1 was released from podocytes, activating EDNRA and promoting mitochondrial oxidative stress and dysfunction, leading to impairment of the endothelial cells (Daehn et al. 2014). In turn, endothelial dysfunction promoted podocyte apoptosis. Conclusively, inhibition of EDNRA prevented podocyte loss, albuminuria, glomerulosclerosis, and renal failure (Daehn et al. 2014).

While it is commonly assumed that podocyte injury precedes endothelial cell injury, Sun et al. demonstrated that in adriamycin-induced nephropathy, endothelial damage occurs prior to podocyte injury (Sun et al. 2013). Finally, Zhang and colleagues investigated serum levels of markers for endothelial dysfunction in patients with primary FSGS and nephrotic range proteinuria, revealing significant higher levels compared with healthy controls (ZHANG et al. 2012). Nevertheless, the role of endothelial cells and their timepoint of pathologic (re-) action will need further intensive studies to reveal potential targets for therapeutic intervention.

## Mesangial cells

In FSGS, mesangial cells characteristically react with expansion, hypercellularity, and increased extracellular matrix deposition. While mesangial expansion is commonly assumed to develop secondary and reactive to, e.g., podocyte loss and PEC activation, isolated studies suggest a more involved role of mesangial cells in FSGS pathogenesis (Strassheim et al. 2013; Jefferson and Shankland 2014). IgM deposits accompanied by deposition of complement factors C3 and C4 in the mesangial compartment of glomeruli were detected in a subset of FSGS biopsies. In contrast to unspecific trapping as an explanation of IgM deposits, the authors hypothesized that an IgM-dependent complement activation contributes to FSGS pathogenesis. Different techniques of B cell depletion in adriamycin-induced glomerulosclerosis led to decreased IgM deposition and an attenuation of albuminuria (Strassheim et al. 2013). Similar results were reported on complement subfragments C4d and C1q potentially even preceding the development of sclerotic lesions (Lest et al. 2019). The precise role of mesangial cells in FSGS and the trigger, which promotes mesangial cell proliferation and extracellular matrix deposition, are not delineated yet and need further examination in order to understand the complete process contributing to FSGS pathogenesis.

## Proposed circulating candidates

A first study in 1984 already reported increased urinary protein excretion when anesthetized rats were perfused with serum of a patient that suffered from recurrent primary FSGS (Zimmerman 1984). In 1999, it was observed that injection of mice with a specific fraction of plasma from recurrent FSGS patients (70% ammonium sulfate supernatant) resulted in proteinuria, while no change was observed after injection of the corresponding plasma fraction from healthy donors (Sharma et al. 1999). These findings indicate the presence of a factor exclusively in recurrent primary FSGS plasma with the ability to induce proteinuria and potentially the recurrence of FSGS after transplantation.

In this section, we will give a brief overview over several factors that have been proposed, but could not be fully confirmed yet by further investigation.

## Active proteinases

Musante et al. identified six proteins purified from serum of children with FSGS which maintained permeability activity in an isolated rat glomeruli assay (Musante et al. 2002). Further characterization of these candidates by mass spectrometry revealed mannan-binding lectin (MBL)-associated serine proteinase as the most likely candidate. In fact, proteinase inhibitors were able to decrease the permeability effect of FSGS sera (Carraro et al. 2004). These results, however, stood in contrast to earlier data, demonstrating no effect of serine proteinase inhibitor phenyl-methylsulfonyl fluoride (PMSF) on reducing albumin permeability activity of FSGS serum (Sharma et al. 1999).

Active proteinases contributing to the pathogenesis of FSGS were again suggested by Harris and colleagues in 2013. Podocytes were incubated with plasma from recurrent primary FSGS patients, resulting in increased phosphorylation of vasodilator-stimulated phosphoprotein (VASP). An increase of VASP phosphorylation was prevented by treatment with protease inhibitors and protease activated receptors (PAR), especially PAR 1, were found to mediate protease-initiated VASP phosphorylation (Harris et al. 2013). Further studies revealed that treatment with supernatant of Th17 cells from healthy controls initiates the same signaling response as PAR1 activation in human podocytes in vitro, leading to a more motile podocyte phenotype and alternated signaling pathways (May et al. 2019)*.* Simultaneous treatment with a protease inhibitor abolished the effect of the Th17 cell supernatant. Based on this, May et al. hypothesized a soluble factor released by Th17 cells, having effects on podocytes similar to PAR-1 activation in vitro (May et al. 2019). The potential factor present in the Th17 cell supernatant and its exact mode of action still needs to be elucidated.

## Cardiotrophin-like cytokine 1

Analysis of the serum of recurrent FSGS patients led to the identification of CLCF-1 by affinity chromatography and mass spectrometry (McCarthy et al. 2010). CLCF-1 belongs to the IL-6 cytokine family and has a predicted molecular weight of 22 kDa (Savin et al. 2012, 2015) with an increase of 100-fold found in FSGS patients (Savin et al. 2012, 2015). Further investigating the effects of recombinant CLCF-1 (rCLCF-1) in vitro showed an effect similar to FSGS serum in vitro and resulted in an increase of glomerular albumin permeability (Savin et al. 2012, 2015) predominantly mediated through the JAK/STAT pathway (Sharma et al. 2015).

## Soluble urokinase-type plasminogen activator receptor

suPAR is the circulating cleaved form of the three domain urokinase-type plasminogen activator receptor (uPAR), a cell membrane glycosylphosphatidylinositol (GPI)-anchored protein, which is expressed on multiple cell types, including immunologically active cells, endothelial cells, and podocytes (Hayek et al. 2015). The first report of uPAR participating in podocyte biology and possibly contributing to podocyte foot process effacement and subsequently proteinuria was published in 2008 (Wei et al. 2008). In vivo experiments in mice revealed avb3 integrin activation in lipid rafts as the link between uPAR signaling induction in podocytes and podocyte foot process effacement, followed by proteinuria (Wei et al. 2008). Analysis of serum samples from FSGS and recurrent FSGS patients revealed significant higher levels of suPAR and circulating suPAR caused foot process effacement and proteinuria in mice (Wei et al. 2011). In a retrospective study suPAR levels, podocyte changes and the effect of treatment on podocyte structure in 25 patients with posttransplant FSGS (recurrent and de novo FSGS) (Alachkar et al. 2013) revealed a positive correlation between suPAR levels and degree of podocyte effacement and treatment with plasmapheresis and in some cases rituximab led to a decrease in suPAR levels (Alachkar et al. 2013). In 2017, immature myeloid cells were proposed to be drivers of proteinuric kidney disease through the production and systemic release of soluble urokinase-type plasminogen activator receptor (suPAR) (Hahm et al. 2017), but the precise role of suPAR in the development of primary FSGS has been questioned in the past.

However, other studies were unable to confirm suPAR as the causative circulating plasma factor in preclinical models (Cathelin et al. 2014; Meijers et al. 2014; Spinale et al. 2014). Elevated serum-suPAR levels were, furthermore, detected in a variety of non-kidney diseases (Cobos et al. 2003; Edsfeldt et al. 2012; Zimmermann et al. 2012) (reviewed in (Maas et al. 2013b)). In kidney diseases, there seems to be a strong correlation between eGFR and serum suPAR levels (exemplified in (Maas et al. 2012) reviewed in (Kronbichler et al. 2016)).

This data points towards a more complex pathophysiology besides the contribution of suPAR to chronic kidney diseases. In accordance, a role of suPAR as a biomarker has emerged in different pathogenic conditions such as in prediction of cardiovascular events (Hayek et al. 2015). Hayek et al. further evaluated the relevance of suPAR in acute kidney injury (Hayek et al. 2020). Plasma suPAR levels in patients with high risk for acute kidney injury, either undergoing coronary angiography or cardiac surgery or critically ill patients on intensive care unit, were correlated with the risk of acute kidney injury, revealing an association between higher suPAR levels and the development of acute kidney injury (Hayek et al. 2020). This association was supported by experiments with suPAR-overexpressing mice, which developed more severe signs of acute kidney injury after injection of contrast material (Hayek et al. 2020).

Taken together, it appears that suPAR could be a contributing factor to kidney injury in general and its levels indicate progression of both proteinuric and non-proteinuric kidney diseases.

## Anti-CD40 antibody

Elevated anti-CD40 antibody levels were detected in sera from recurrent FSGS patients and their capability of predicting the recurrence of FSGS before transplantation was evaluated (Delville et al. 2014). CD40 is a costimulatory transmembrane receptor, which belongs to the tumor necrosis factor gene superfamily (Chatzigeorgiou et al. 2009) mainly expressed on the surface of antigen presenting cells (APC) but is also found on a variety of other cell types, e.g., epithelial cells. Recently, CD40 is known to participate in a variety of immunological processes, including activation and further differentiation of immune cells as well as production and secretion of different cytokines and chemokines (Chatzigeorgiou et al. 2009).

Elevated anti-CD40 antibodies predicted a recurrence of FSGS with an accuracy of 78% (Delville et al. 2014). Incubation of podocytes in vitro, both with recurrent FSGS sera and purified anti-CD40-antibodies from recurrent FSGS patients resulted in podocyte depolarization and reduction of overall cell size with decrease of cytoskeletal F-actin expression and injection of anti-CD40-antibody from recurrent FSGS patients in mice led to a mild but significant increase in urinary protein excretion (Delville et al. 2014). Anti-CD40 antibodies being a main pathogenic factor in FSGS are in contrast to previous studies, which suggested a smaller protein fraction (30–50 kDa) to contain the pathogenic plasma intrinsic factor, as immunoglobulins have a size of 150 kDa (Sharma et al. 1999; Vidarsson et al. 2014).

## Therapeutic targets

A plethora of unknown variables in the pathogenesis of primary and recurrent FSGS challenges the clinical decision on individually appropriate therapies.

Patients with primary FSGS usually receive nephroprotective-based therapy, including renin-angiotensin-aldosterone system (RAAS)-blockade with either angiotensin converting enzyme inhibitors (ACEi) or angiotensin II receptor blockers (ARBs). Further treatment includes lifestyle changes, like nicotine cessation, weight reduction, and low sodium diet (KDIGO KGW 2012). Supplemental therapy consists of prescription of statins.

Immunosuppressive therapy is usually indicated in the presence of nephrotic range proteinuria. The commonly prescribed immunosuppressive drug is prednisone, which is recommended in a dose of 1 mg/kg bodyweight per day or 2 mg/kg bodyweight on alternating days (KDIGO KGW 2012). In steroid-resistant cases, the addition of Cyclosporin A (CsA) to the therapy regimen is warranted. In case of failure of the aforementioned therapies, no consensus about the further therapeutic procedure exists. Ensuing treatment relies on center-based decisions and is mostly based on empiric trials and clinicians’ experience. Even a bigger challenge displays the therapy of recurrent FSGS in kidney allografts. Patients after kidney transplantation, already receiving immunosuppressive therapy and nonetheless developing recurrent FSGS, are particularly challenging to treat. Several treatment opportunities have emerged in the past considering different approaches to affect FSGS activity and rely either on attenuation of the immunological response, removal of causative factors from the patients’ blood, or on interference of pathological pathways in FSGS.

One alternative treatment option, based on the beneficial effect of prednisone in many FSGS patients, is the application of ACTH gel. ACTH gel is proposed to have an antiproteinuric effect via melanocortin 1 receptor, thus exerting beneficial effects additional to those mediated by corticosteroids (Alhamad et al. 2019; Grafals and Sharfuddin 2019).

Also, targeted therapies find application in treatment of primary and recurrent FSGS. The mouse/human chimeric CD20 antibody rituximab is frequently used in several autoimmune diseases and has also been demonstrated to be successful in FSGS treatment (Leandro et al. 2002; Edwards et al. 2004; Nozu et al. 2005; Pescovitz et al. 2006; Hauser et al. 2008; Alasfar et al. 2018).

Ofatumumab, another CD20 antibody with higher affinity to CD20 and an alternating epitope binding, was also reported in separate case reports to have a beneficial effect on remission in recurrent FSGS patients with resistance to other therapies (Kienzl-Wagner et al. 2018; Colucci et al. 2020).

Furthermore, suggestions of tumor necrosis factor α (TNFα) being involved in the pathogenesis of FSGS and its recurrence, raised interest in anti-TNFα antibodies (Infliximab, Adalimumab) as a therapeutic option (Trachtman et al. 2015; Otalora et al. 2019).

Complementing the group of targeted therapies, the fusion proteins Abatacept and Belatacept, inhibitors of the co-stimulation molecule B7-1, showed very promising results in an initial study by Yu et al. (2013). However, attempts to reproduce these results by other groups failed, leading to discontinuation of Abatacept and Belatacept application in recurrent FSGS patients (Alachkar et al. 2014; Grellier et al. 2015; Delville et al. 2015).

Upon the hypothesis of galactose neutralizing the causative factors and thus reducing FSGS activity, clinical trials with intravenous and/or oral galactose application were initiated and cases with successful galactose treatment have been reported (Savin et al. 2008; Robson et al. 2015; Trachtman et al. 2015). Next to steroids and targeted therapies, extracorporeal strategies play an important role in treatment of primary and recurrent FSGS. These strategies include plasmapheresis (PP)/therapeutic plasma exchange (TPE), immunoadsorption (IA) and low-density-lipoprotein (LDL) apheresis to separate the triggering factor from the blood (Ohta et al. 2001; Kobayashi 2008; Schwartz et al. 2013; Lionaki et al. 2015; Muso et al. 2015; Kashgary et al. 2016; Allard et al. 2017; Shah et al. 2019).

## Limitations and future directions

**Fig. 1:**
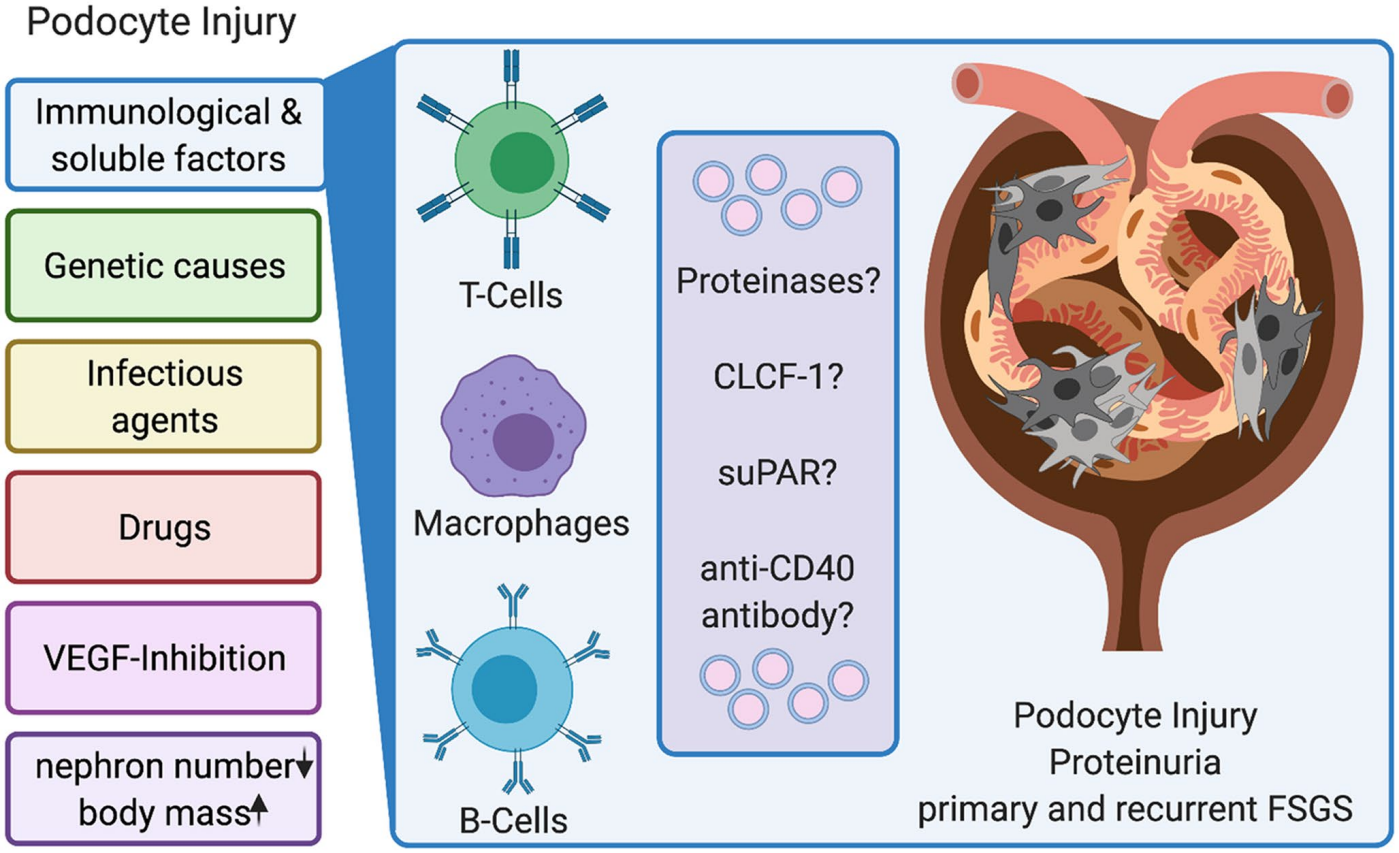
Entities of podocyte injury besides systemic effects and proposed mechanisms in primary FSGS. Created with BioRender.com

Despite the extensive research in the field of primary and recurrent FSGS for decades and steady progress in delineating individual mechanisms and factors contributing to the initiation and progression, there is no definitive understanding of the pathogenesis of primary and recurrent FSGS today (Fig. 1). Preclinical models offer first evidence for the cellular effectors and recipients driving the development of pFSGS, but no current model can concisely mirror the clinical course observed in patients. At the same time, the majority of clinical studies relied on mixed cohorts of not clearly defined FSGS entities and a potential overlap of common causes of podocyte depletion and sclerosis development. To this day, none of the proposed soluble factors could be validated in a clinical setting by removing the potential permeability factor(s) from the patients’ blood.

Better stratified patient cohorts and exploiting the recent developments in deep (single cell) molecular analysis of the immune cell-podocyte interaction network will hopefully reveal the missing pieces in this most enigmatic nephrology puzzle in the next decade.

## Declarations

### Research involving human and animal participants

This article does not contain any studies with human participants or animals performed by any of the authors.
